# Acute Response of Sclerostin to Whole-body Vibration with Blood Flow Restriction

**DOI:** 10.1055/a-1422-3376

**Published:** 2021-05-11

**Authors:** Kyle S Gapper, Sally Stevens, Rona Antoni, Julie Hunt, Sarah J Allison

**Affiliations:** 1 Department of Bioscience & Medicine, University of Surrey, Guildford, United Kingdom of Great Britain and Northern Ireland

**Keywords:** bone metabolism, bone turnover markers, blood flow restriction, vibration, acute response

## Abstract

Blood flow restriction may augment the skeletal response to whole-body vibration. This study used a randomised, crossover design to investigate the acute response of serum sclerostin and bone turnover biomarkers to whole-body vibration with blood flow restriction. Ten healthy males (mean±standard deviation; age: 27±8 years) completed two experimental conditions separated by 7 days: (i) whole-body vibration (10 1-minute bouts of whole-body vibration with 30 s recovery) or (ii) whole-body vibration with lower-body blood flow restriction (10 cycles of 110 mmHg inflation with 30 s deflation during recovery). Fasting blood samples were obtained immediately before and immediately after exercise, then 1 hour, and 24 hours after exercise. Serum samples were analysed for sclerostin, cross-linked C-terminal telopeptide of type I collagen, and bone-specific alkaline phosphatase. There was a significant time × condition interaction for bone-specific alkaline phosphatase (p=0.003); bone-specific alkaline phosphatase values at 24 hours post-exercise were significantly higher following whole-body vibration compared to combined whole-body vibration and blood flow restriction (p=0.028). No significant time × condition interaction occurred for any other outcome measure (p>0.05). These findings suggest that a single session of whole-body vibration combined with blood flow restriction does not significantly affect serum sclerostin or bone turnover biomarkers.

## Introduction

Blood flow restriction (BFR) involves the application of a tourniquet system to the proximal portion of a limb to partially restrict arterial inflow and fully restrict venous outflow 1 . Mechanical compression of the underlying vasculature induces localised hypoxia distal to the placement of the cuff while causing blood pooling within the capillaries of the occluded limbs via diminution of venous blood flow 2 . This technique has typically been applied to resistance exercise to augment the muscle hypertrophic and strength responses to low-load resistance training 1 3 4 . However, there is emerging evidence to suggest that BFR exercise may also provide a novel stimulus to bone.

Bone turnover markers are proteins or degradation products released during the process of bone remodelling and include markers of bone formation and bone resorption 5 . Bone turnover markers have been associated with bone mineral density (BMD) 6 7 and fracture risk 8 and have been used to evaluate exercise and training effects 9 . Some studies have investigated the response of bone turnover markers following BFR exercise 10 11 12 . BFR increased the expression of bone formation biomarkers such as bone-specific alkaline phosphatase (B-ALP) and/or decreased bone resorption biomarkers such as the cross-linking telopeptides of type I collagen (CTX) when combined with walking or resistance exercise 10 11 12 . Interestingly, one study reported a reduction in CTX 30 minutes following low-load BFR resistance exercise that was of a greater magnitude than the reductions reported following unrestricted low-load resistance exercise 11 . Although these prior findings are encouraging, the application of BFR to date has been limited to walking or resistance exercise and the acute osteogenic response of BFR exercise has only been assessed by markers of bone formation and resorption.

Osteocytes are the primary bone cells that sense mechanical strain via fluid flow shear stress through the lacuna-canalicular network and changes in interstitial hydrostatic pressure 13 14 15 . One potentially important mechanism by which mechanical stimuli influence osteocyte activity is through regulating sclerostin, an osteocyte-derived secreted glycoprotein, which inhibits bone formation through inhibition of the Wnt/β-catenin pathway 16 17 . There have been few human studies to date reporting the response of sclerostin to acute exercise, although current evidence suggests that sclerostin levels increase following an acute bout of physical activity 18 19 , high-intensity interval exercise 20 , jumping 21 , whole-body vibration (WBV) 22 , and combined high-intensity resistance training and WBV 23 .

BFR applied to WBV could provide a new mode of stimulation to optimise bone health in populations that may have difficulty performing high-impact or high-intensity resistance exercise, such as untrained individuals, older adults, or those undergoing musculoskeletal rehabilitation. WBV provides mechanical stimuli to bone in the form of vertical oscillation, which is adequate to increase fluid flow in bone and produce an osteogenic signal 24 . Since elevations in intramedullary pressure and changes in bone fluid flow is one of the mechanisms through which BFR can stimulate bone adaptation 25 , adding a BFR stimulus to a traditional WBV protocol may potentially exert an additive osteogenic effect. However, the response of sclerostin and bone turnover makers to combined WBV and BFR is yet to be explored. Such investigations could provide insight into the specificity of the bone response to BFR exercise in the short term. The primary aim of this study was to investigate the response of serum sclerostin and bone turnover biomarkers to an acute bout of WBV superimposed with BFR. It was hypothesised that combined WBV and BFR would transiently increase serum sclerostin concentrations to a greater extent than that resulting from WBV alone.

## Materials and Methods

### Participants

Ten healthy untrained males aged 18–39 years volunteered to participate in this investigation (see Table 1 for participant demographics). Participants were excluded from the study if they met any of the following exclusion criteria: (i) current smoker; (ii) a BMI ≥ 30 kg/m ^2^ ; (iii) a history of cardiovascular (including hypertension), metabolic, haematological, neurological, or musculoskeletal disease or injury; (v) consumed medication or supplements known to influence bone status; and (vi) performed weight-bearing endurance or resistance training more than three times per week over the preceding 12 months. Before initiating the study, all participants provided written informed consent and completed a health history questionnaire to document information about exercise training status and to detect potential risk factors that may be aggravated by performing the type of activity in the study. Participants also completed the bone-specific physical activity questionnaire (BPAQ) to quantify the degree of activity-related skeletal loading from current (previous 12 months) and past (from one year of age) activities 26 . This investigation was granted a favourable ethical opinion from the University of Surrey Ethics Committee and conformed to the ethical requirements stipulated by the International Journal of Sports Medicine 27 .

**Table:** **Table 1** Participant demographics ( *n* =10).

	Mean±SD
Age (years)	27±8
Body mass (kg)	78.5±9.4
Height (cm)	179.2±5.2
BMI (kg/m ^2^ )	24.5±2.8
Resting blood pressure (mmHg)	
Systolic blood pressure	129±8
Diastolic blood pressure	71±8
BPAQ scores	
Current BPAQ score	6.1±5.9
Past BPAQ score	39.2±30

Note: BMI, body mass index; BPAQ, bone-specific physical activity questionnaire.

### Experimental design

**Fig. 1:**
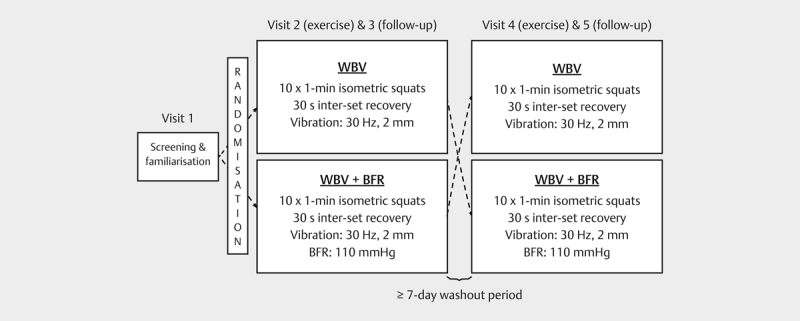
Schematic overview of the study design. WBV, whole-body vibration; WBV+BFR, whole-body vibration with blood flow restriction.

A randomised, crossover design was used to investigate the acute response of serum sclerostin and bone turnover biomarkers to a single session of WBV exercise with and without BFR. Participants attended the laboratory on five separate occasions (familiarisation, two experimental trials, and two 24-h follow-up visits), with each experimental trial separated by a minimum of 7 days. A schematic overview of the study design is presented in Fig. 1 .

During the first visit to the laboratory, participants completed all paperwork, provided measures of height (Seca 220; Seca, Hamburg, Germany), body mass (Seca 761; Seca), and blood pressure (Omron M2; OMRON Healthcare, Kyoto, Japan), and were familiarised with the exercise equipment and protocol. Participants then attended the laboratory on four more occasions to complete two experimental conditions in a random counterbalanced order: (i) whole-body vibration only (WBV), and (ii) whole-body vibration with blood flow restriction (WBV+BFR). Each experimental trial had a follow-up visit 24 hours later. Participants attended and completed all experimental trials and follow-up visits having undertaken an overnight fast of a minimum of 8 hours. Each exercise bout was performed at the same time of day. Participants recorded their dietary and activity habits during the 72 hours preceding the first experimental trial and were asked to replicate these habits before the second trial. Total energy, macronutrient, calcium, and vitamin D intake were quantified using nutrition analysis software (Nutritics LTD, Dublin, Ireland). Participants abstained from strenuous physical activity, caffeine, and alcohol consumption for at least 72 hours before each visit.

### Loading protocols

**Fig. 2:**
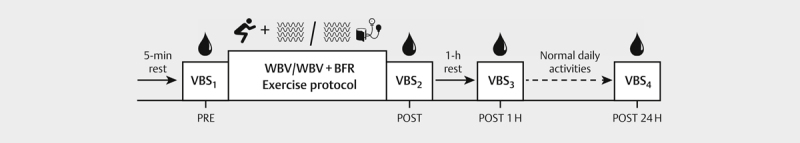
Schematic overview of the experimental procedures for the study. WBV, whole-body vibration; WBV+BFR, whole-body vibration; VBS _1–4,_ venous blood sample 1–4; PRE, pre-exercise; POST, immediately post-exercise; POST 1H, 1-hour post-exercise; POST 24H, 24 hours post-exercise.

A schematic overview of the experimental protocol is presented in Fig. 2 .

#### Whole-body vibration (WBV)

Participants underwent ten 1-minute bouts of vibration exercise with 30 s recovery between repetitions; the total duration of vibration was 10 minutes. Participants stood barefoot on a synchronous vibration platform (Power Plate NEXT generation; Performance Health Systems UK Ltd., London UK) in an isometric semi-squat position with a relative knee joint angle of 130°. The knee joint angle was measured manually using a goniometer to maintain consistency between trials. Participants performed each squat with their hands on their hips and were instructed to keep balanced foot plantar pressure between the rear- and forefoot. A semi-squat position with static posture was chosen as it has been shown to improve bone mineral density when performed with WBV while minimising transmission to the head 28 . The vertical vibration stimulus was administered at a frequency of 30 Hz with a peak-to-peak displacement (i. e. displacement from the lowest to the highest point of the total vibration excursion) of 2 mm as reported by the manufacturer. A “low” setting was used on the Power Plate to achieve the desired vibration amplitude. Expressed as a multiple of Earth’s gravity, the peak acceleration of the vibration stimulus was 3.6 g based the equation by Rauch et al. 29 . This vibration intensity (e. g. frequency and peak-to-peak amplitude) is similar to that used in other acute studies that have documented positive physiological changes during and following combined WBV and BFR 30 31 .

#### Whole-body vibration with blood flow restriction (WBV+BFR)

The WBV+BFR condition used the WBV protocol described above with the addition of lower-limb BFR. A 13-cm-wide pneumatic cuff (Model SC12LTM; Hokanson, Bellevue, WA, USA) was applied to the most proximal portion of each thigh (distal to the inguinal fold) and inflated (E20 Rapid Cuff Inflator and AG101 Cuff Inflator Air Source; Hokanson) to 110 mmHg for the entire 1-minute duration of each repetition. Both thigh cuffs were fully deflated during each 30-second recovery interval. The selected inflation pressure was similar to earlier work 32 showing that the absolute pressure required to occlude lower-body arterial blood flow is approximately 40% lower when using wider cuffs compared to narrow cuffs in a sample of healthy, young adults. At 110 mmHg, we estimate that the degree of blood flow restriction attained was approximately 60% 33 . An intermittent BFR protocol (i. e. cuff pressure released between exercises/exercise sets) was selected based on longer-term studies documenting its ability to influence bone changes 34 35 . Our decision to use short inflation periods draws support from prior evidence that bone responds to mechanical stimulus that is brief and interspersed with short rest periods 36 . It is also important to note that in pilot testing, participants were unable to complete the desired exercise volume with the cuff inflated throughout, so it was not practical to use a continuous BFR protocol at this pressure.

### Blood sampling

Venepuncture blood samples from both experimental trials were collected between 07:00 and 09:00 hours and the time of blood draw was consistent for each participant. Samples were obtained by a qualified phlebotomist before (PRE), immediately after (POST), 1-hour after (POST 1H), and 24 hours after (POST 24H) the exercise session. Each blood sample was drawn from an antecubital vein and gathered into 4-ml serum separator tubes. Collected samples were allowed to clot at room temperature for 30 minutes. Serum was separated by centrifugation at 2500 rpm for 10 minutes, and serum aliquot was frozen at –20° until the assay analyses were performed. All samples were analysed in batch upon trial completion.

### Bone biomarker analysis

Serum sclerostin was determined using the TECO Medical high-sensitivity enzyme immunoassay (EIA) (Quidel Corporation, San Diego, CA, USA). Serum CTX and B-ALP were assessed using an enzyme-linked immunosorbent assay (ELISA) (CTX: Immunodiagnostic Systems, Tyne & Wear, UK; B-ALP: MicroVue BAP EIA; Quidel Corporation). The intra- and inter-assay coefficient of variation for sclerostin, CTX and B-ALP ranged between 3.7–4.2% and 4.3–4.8%, 1.7–3.0% and 2.5–10.9%, 3.9–5.8% and 5.0–7.6% respectively, as stated by the manufacturer. All samples were run in duplicate and had undergone a single freeze/thaw cycle.

### Statistical analysis

Statistical analyses were performed using IBM SPSS Statistics 25.0 for Windows (IBM Corp., Armonk, NY, USA). Normality of the data set was evaluated using the Shapiro-Wilk test. Between-trial differences in overall energy intake and protein, carbohydrate, calcium, and vitamin D consumption were assessed using paired-samples t-tests. A two-way (condition × time) repeated-measures analysis of variance (ANOVA) was used to determine the effects of the experimental conditions over time on serum sclerostin, CTX, and B-ALP concentrations. Mauchly’s test of sphericity was conducted to assess for sphericity of data, and Greenhouse-Geisser adjustments were made to correct for sphericity violations. Any significant main effects of time were assessed using separate one-way repeated measures ANOVAs. When significant condition × time interactions occurred, paired-samples t-tests with a Bonferroni correction for multiple comparisons were conducted at each level of time to locate the time point at which significant effects were present.

Effect sizes were computed as Cohen’s d or partial eta-squared (η _p_^2^ ). Cohen’s d was calculated as the mean difference of the two values at a given time point divided by the pooled SD of the two values 37 . Partial eta-squared was calculated as the sum of squares of the effect divided by the sum of squares of the same effect and its associated error variance 38 . All data are presented as mean±95% confidence intervals unless otherwise stated. Statistical significance was accepted at p<0.05.

## Results

### Participant demographics

Participant demographics are summarised in Table 1 .

### Dietary intake

Overall energy intake (p=0.48) and protein (p=0.78), carbohydrate (p=0.53), calcium (p=0.32), and vitamin D (p=0.14) consumption were similar over the 72 hours preceding the two experimental trials. Descriptive food diary data are presented in Table 2 .

**Table:** **Table 2** Comparison of energy and nutrient intake data derived from food diaries completed over the 72 hours preceding both experimental trials ( *n* =10).

	WBV	WBV+BFR	p-value
	Mean±SD	Mean±SD	
Energy intake (kJ)	8121±1702	8355±2204	0.48
Carbohydrate (g)	221±39	213±57	0.53
Protein (g)	91±34	92±29	0.78
Calcium (mg)	966±243	1096±396	0.32
Vitamin D (µg)	1.9±1.3	2.6±1.7	0.14

Note: WBV, whole-body vibration; WBV+BFR, whole-body vibration with blood flow restriction. P-value indicates no between-trial differences assessed using paired-samples t-test.

### Bone biomarker responses

An overview of all bone biomarker response data is provided in Table 3 .

**Table:** **Table 3** Descriptive data for all bone biomarker responses measured before and after whole-body vibration exercise with and without blood flow restriction (n=10).

	WBV				WBV+BFR			
	PRE	POST	POST 1H	POST 24H	PRE	POST	POST 1H	POST 24H
**Sclerostin (ng·mL** ^**−1**^ **)**	0.418±0.193	0.460±0.240	0.448±0.251	0.433±0.199	0.404±0.205	0.425±0.192	0.403±0.162	0.439±0.186
95% CI (ng·mL ^−1^ )	0.281–0.556	0.289–0.632	0.268–0.628	0.291–0.575	0.257–0.550	0.288–0.562	0.287–0.519	0.304–0.574
Change from PRE (%)	–	9.9±17.8	7.1±19.6	5.3±19.3	–	9.8±22.2	9.5±33.2	17.7±32.0
**CTX (ng·mL** ^**-1**^ **)**	0.610±0.452	0.598±0.415	0.529±0.350	0.648±0.450	0.566±0.337	0.535±0.307	0.504±0.333	0.514±0.299
95% CI (ng·mL ^-1^ )	0.287–0.933	0.301–0.895	0.278–0.779	0.326–0.969	0.325–0.806	0.316–0.755	0.265–0.742	0.300–0.728
Change from PRE (%)	–	0.9±16.0	−10.9±15.7	6.1±20.6	–	−3.3±18.2	−11.7±22.5	−7.7±24.5
**B-ALP (μL)**	23.35±7.64	24.18±7.71	24.87±8.10	25.94±8.82 *	24.42±9.07	25.35±8.14	24.90±9.10	23.47±8.29
95% CI (μL)	17.89–28.82	18.66–29.70	19.08–30.66	19.63–32.25	17.93–30.90	19.52–31.17	18.39–31.42	17.54–29.40
Change from PRE (%)	–	4.1±6.0	6.8±5.2	11.0±6.8	–	6.0±9.2	2.5±8.7	−3.4±2.4

Note: Data are reported as mean±SD. WBV whole-body vibration, WBV+BFR whole-body vibration with blood flow restriction, CTX cross-linked C-terminal telopeptide of type 1 collagen, B-ALP bone-specific alkaline phosphatase, PRE pre-exercise, POST immediately post-exercise, POST 1H 1-hour post-exercise, POST 24H 24 hours post-exercise, 95% CI 95% confidence intervals. * Indicates a significant between-condition difference (p < 0.05).

#### Sclerostin

**Fig. 3:**
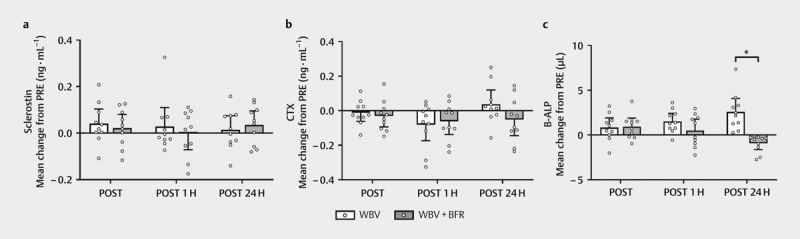
Mean absolute change of **a** serum sclerostin, **b** serum cross-linked C-terminal telopeptide of type 1 collagen (CTX), and **c** serum bone-specific alkaline phosphatase (B-ALP) from pre-exercise values following whole-body vibration exercise (WBV) and whole-body vibration exercise with blood flow restriction (WBV+BFR). Bars represent mean±95% confidence intervals, and open symbols are individual data ( *n=* 10). POST, immediately post-exercise; POST 1H, 1-hour post-exercise; POST 24H, 24 hours post-exercise ^*^ Indicates a significant between-condition difference (p<0.05).

There was no statistically significant main effect of condition (p=0.14; η _p_^2^ =0.22) or time (p=0.43; η _p_^2^ =0.08), and no significant condition × time interaction (p=0.68; η _p_^2^ =0.05) for serum sclerostin. The absolute mean change of serum sclerostin from pre-exercise values is presented in Fig. 3a .

#### CTX

A significant main effect of time (p=0.018; η _p_^2^ =0.31) but not condition (p=0.17; η _p_^2^ =0.20) was revealed for serum CTX. No condition × time interaction occurred (p=0.27; η _p_^2^ =0.13). Despite indications of a significant main effect of time, post-hoc one-way ANOVAs revealed no significant difference between time points for either condition (p>0.05). The absolute mean change of serum CTX from pre-exercise values is presented in Fig. 3b .

#### B-ALP

There was no significant main effect of condition (p=0.94; η _p_^2^ =0.001) or time (p=0.086; η _p_^2^ =0.24) for serum B-ALP; however, there was a significant condition × time interaction (p<0.001; η _p_^2=^ 0.49). Paired-samples t-tests with Bonferroni corrections revealed that serum B-ALP was significantly greater in WBV compared to WBV+BFR at POST 24H (p=0.028, *d* =0.31). The absolute mean change of serum B-ALP from pre-exercise values is presented in Fig. 3c .

## Discussion

The primary aim of this study was to investigate the responses of sclerostin and bone turnover biomarkers to an acute bout of WBV superimposed with BFR. To achieve this aim, we controlled dietary intake and physical activity before test days and evaluated serum concentrations of sclerostin and biomarkers of bone turnover at periodic time points up to 24 hours after exercise. In contrast to our hypothesis, we found no significant changes in sclerostin or biomarkers of bone turnover in response to a single session of blood flow-restricted WBV exercise.

Few human studies have examined changes in sclerostin in response to exercise. A transient increase in serum sclerostin has been reported following an acute bout of WBV 22 , combined resistance training with WBV 23 , physical activity 18 19 , high-intensity interval exercise 20 , and jumping 21 . One study found a lack of change in sclerostin immediately following five 1-minute bouts of WBV (20 Hz, 3.38 mm, ~2.7 g) 23 , whereas another study reported a 91% increase in plasma sclerostin from pre- to 10 minutes post-WBV (40 Hz, 2 mm, ~2.7 g) 22 . The contrast in findings may be attributed to the differences in the time point analyses or factors relating to the vibration stimulus. Bone cellular responses to mechanostimulation are influenced by the specific components of the imposed stressor 39 . Thus, between-study disparities in vibration variables such as frequency (30 Hz versus 40 Hz for the present study and Cidem et al. 22 , respectively) may explain the differential sclerostin response to WBV. It is worth noting that, based on the peak acceleration equation by Rauch et al. 29 , the vibration stimulus applied in the study by Cidem et al. 22 may actually equate to an acceleration level of 6.4 g, rather than the 2.7 g reported. This may suggest that the sclerostin response to WBV exercise is largely governed by the overall magnitude of the vibration stimulus. Further research is needed to determine the influence of the various vibration parameters (frequency, displacement, acceleration, and dose) on serum sclerostin over an extended time course.

Interestingly, we found that serum B-ALP values at 24 hours post-exercise were significantly greater following WBV compared to WBV+BFR. The acute rise in serum B-ALP 24 hours following WBV is suggestive of a favourable bone metabolic shift towards enhanced bone formation, particularly when considered alongside a non-significant change in CTX. To our knowledge, no other study has examined the response of B-ALP to WBV for up to 24 hours post-exercise. When combined with resistance exercise, previous studies have shown that B-ALP either increases or remains unchanged 30 minutes post-exercise 40 41 . It is possible that a longer time-window may be required to detect changes in serum B-ALP in response to WBV. In support of this, other studies have found significant increases in the bone formation marker osteocalcin following WBV, which peaked at 48 hours post-exercise 42 .

No additive effect of BFR on the response of B-ALP or CTX to WBV was observed at any time point. These findings are likely due to components of the BFR stimulus (e. g. frequency, cuff size, fixed cuff pressure, restriction time, intermittent protocol) not being optimal to elicit bone cellular responses. One variable that may explain our lack of significant findings is the magnitude of BFR attained during exercise. Acute and chronic training studies that have shown BFR-induced changes in CTX and B-ALP have typically used elastic cuffs 5 cm in width with absolute cuff pressures in the range of ~160–230 mmHg 10 11 12 . In the present study, we used a cuff 13 cm wide and an absolute cuff pressure of 110 mmHg, as the cuff pressure required to occlude lower-body arterial blood flow has been reported to be approximately 40% lower when using wider cuffs compared to narrow cuffs 32 . Notably, another BFR study utilising a similar study population and cuff width (13.5 cm) reported that a cuff pressure of 139.75±14.41 mmHg was enough to occlude lower-body blood flow 43 . Current literature suggests that the cuff pressure required to occlude blood flow to a limb (i. e. arterial occlusion pressure; AOP) is dependent on cuff properties (e. g. tourniquet shape, width, and length) and individual blood pressure and limb characteristics. Hence, it is suggested that cuff pressures should be set to a percentage of an individual’s AOP, with cuff pressures equating to 50–80% of AOP typically recommended to support the efficacy of BFR training 2 . Based on previous observations, we estimate that our selected restriction pressure of 110 mmHg restricted lower-limb blood flow by ~60% 33 . This considered, it is possible that a cuff pressure of 110 mmHg was not high enough to elicit any bone cellular responses. In addition, a fixed BFR pressure may result in different levels of BFR within the sample of the study, which may, consequently, reflect distinct exercise-induced physiological responses and therefore increase variability. Future studies should consider setting cuff pressure as a relative percentage of an individual’s AOP to standardise restriction pressure across participants and mitigate inter-individual variability in BFR-induced physiological responses. Future research should also seek to clarify if different restriction pressures are requisite for stimulating bone cellular responses taking into consideration the discomfort and safety of the cuff pressure in the long-term.

Several physiological mechanisms by which BFR training may influence bone metabolism and evoke chronic skeletal adaptations have been proposed, including increased intramedullary pressure and interstitial fluid flow within the bone during BFR 44 . Additionally, sustained BFR-induced hypoxia may upregulate bone remodelling-related genes and induce downstream changes in bone cellular activity 45 . One factor that may influence the magnitude of localised hypoxia distal to the site of the cuff is the restriction time. Previous studies that have reported BFR-induced changes in bone turnover biomarkers have used continuous 10 11 and intermittent BFR protocols 12 , and a minimum restriction time of 6–15 minutes per exercise. In contrast, the present study utilised an intermittent BFR protocol of ten cycles of 1 minute inflation/30 seconds deflation. Therefore, the chosen restriction time may not have been of sufficient duration to create any meaningful degree of localised hypoxia. A restriction time of a minimum of 5 minutes per exercise is recommended to promote improvements in muscle strength and hypertrophy 2 , although no such guidelines for skeletal adaptations currently exist. Based on the conflicting findings between our study and others, it is possible that longer restriction times may be required to promote bone cellular responses. Prolonged restriction times would likely extend the duration of the acute state of hypoxia in the tissues below the cuff, possibly causing alterations of bone bioenergetics and upregulation of bone remodelling-related genes through the stabilisation of hypoxic-induced gene transcription factors (HIFs) and activation of the HIF pathway 46 .

The decision to apply an intermittent BFR protocol with short restriction times was informed by prior findings that a prolonged mechanical stimulus leads to an exponential reduction in bone mechanosensitivity that diminishes the osteogenic response to loading 36 . Intermittent mechanical loading interspersed with rest periods has been reported to elicit greater bone responses than continuous mechanical loading in animal models 47 48 49 , potentially due to the inclusion of brief rest intervals that allow the bone to “resensitise” to the loading stimulus 50 . Our rationale for deflating the cuffs during the rest period was an attempt to mimic cyclic loading that has been reported to influence fluid flow and intramedullary pressure sensed by osteocytes that release sclerostin 51 . However, this may have been a limiting factor in being able to detect a difference between the two protocols. Previous research has shown the addition of BFR to an acute bout of WBV increases myoelectric activity 52 53 , metabolite accumulation 52 53 , and serum levels of growth hormone 53 to a greater extent than WBV exercise alone when the cuff pressure was inflated during rest periods. Since muscle activity and hypoxia-driven physiological changes may influence bone metabolism 54 55 , future studies may consider keeping the cuff pressure inflated during the entire exercise including rest periods, yet also consider the discomfort of the cuff pressure.

This study is novel in that it is the first study to investigate the acute response of serum sclerostin and biomarkers of bone turnover to WBV with BFR. The strengths of this study include the crossover design with random assignment of WBV or combined WBV and BFR. To account for the circadian variation in bone biomarkers 56 57 , the exercise protocol and venous blood sampling were performed at the same time of day for both experimental conditions, and we measured the bone biomarker response up to 24 hours after exercise. However, this study also has several limitations that must be considered when interpreting the findings. Firstly, our study population consisted of untrained but otherwise healthy adult males so the present findings should not be extrapolated to other study populations, particularly those subject to skeletal weakness, such as postmenopausal women and older adults. Secondly, the semi-squat position was standardised by a goniometric assessment of knee joint angle, but other biomechanical parameters such as joint angle at the hip and ankle may influence the transmission of the vibration stimulus. Thirdly, we did not include a BFR-only control condition that would have provided insight into the overall contribution of the BFR stimulus to the reported bone biomarker responses. Fourthly, we used B-ALP and CTX as proxy measures of bone formation and resorption, respectively, in line with similar studies that have investigated the acute bone turnover response to exercise 11 40 41 . Future clinical investigations into the response of bone turnover to BFR strategies should consider using recommended biomarkers of bone formation, such as N-terminal propeptide of type 1 procollagen (P1NP) 58 . Finally, while bone biomarkers are commonly used to provide a "snapshot" of the bone turnover response to exercise, they are affected by various endogenous and exogenous sources of variability, including pre-exercise feeding and loading history, that may mask the true dynamics of bone metabolism 59 . To mitigate this, we asked participants to replicate their diet and activity during the 72 hours leading up to the experimental trials, but more rigorous standardisations could be considered in future studies.

In conclusion, the present findings suggest that the addition of BFR to ten 1-minute bouts of WBV elicited no significant change in the serum concentration of sclerostin, CTX, or B-ALP at any timepoint up to 24 hours post-exercise in untrained, adult men. Future investigations into the effects of combined WBV and BFR exercise on bone metabolism should consider the parameters and overall intensity of the vibration stimulus, the methodological factors that influence BFR such as standardisation of restriction pressures while considering the discomfort and safety of the cuff pressure, and include populations at risk of skeletal weakness such as postmenopausal women. Further efforts should be made to fully elucidate the osteogenic potential of BFR, and whether it may be beneficial to perform in conjunction with other exercise modalities.
